# Knowledge about Avian Influenza, European Region 

**DOI:** 10.3201/eid1412.080858

**Published:** 2008-12

**Authors:** Elias Mossialos, Caroline Rudisill

**Affiliations:** London School of Economics, London, UK

**Keywords:** Avian influenza, bird flu, Europe, letter

**To the Editor:** Since the first identifications of avian influenza (H5N1) in Europe in late 2005 and early 2006, Eurobarometer survey data obtained during April–May 2006 have provided a unique opportunity to examine the knowledge of respondents across the European Union, Croatia, and Turkey about the risks and transmission of avian influenza. The H5N1 strain of avian influenza virus has caused >240 human deaths in central and Southeast Asia, the Middle East, and Africa (*1*). Four of these deaths occurred in Turkey in 2006. Understanding gaps in the public’s knowledge about avian influenza risks and transmission provides guidance on which issues future public health information campaigns may wish to focus. From a public health perspective, a more informed general public will be less likely to unnecessarily alter their travel and food consumption behavior and more likely to take appropriate preventive actions.

A 2006 Eurobarometer survey asked 29,170 residents of the 27 countries in the European Union, Croatia, and Turkey about their knowledge of avian influenza risks (*2*). Eurobarometer surveys are undertaken by the European Commission to monitor the EU public’s social and political opinions. The survey was conducted on a multistage random sampling basis. Therefore, the sample is representative of the whole territory surveyed. Each country’s population was randomly sampled according to rural, metropolitan, and urban population densities. A cluster of addresses was selected from each primary sampling unit by using country-dependent resources such as electoral registers. Addresses were chosen systematically by using standard random route procedures, beginning with a randomly selected initial address. The survey was conducted by face-to-face interviews in respondents’ homes.

Data were collected from March 27 through May 1, 2006. This period is especially interesting when looking at Europeans’ knowledge about avian influenza risk because the first European cases of avian influenza (H5N1) were found in October 2005 in Turkey; additional cases were found later that month in Romania, Croatia, and the United Kingdom. Therefore, the period would have included media coverage about avian influenza as well as any targeted public health efforts to inform residents about avian influenza risks. By the end of this survey’s fieldwork period, 17 of the 29 countries surveyed had reported influenza virus (H5N1) in birds, 3 in mammals, and 1 in humans (*3*).

Respondents were asked 7 questions about their knowledge of the risks humans face regarding avian influenza (Table). When we looked at these results with the aim of setting future public health information campaign objectives, we considered incorrect or “don’t know” responses to indicate public health information campaign failures. Uncertainty regarding avian influenza risks appeared to involve consumption of eggs and vaccinated, cooked poultry and whether the virus can be transmitted between humans. However, for all questions asked, more than half of the respondents answered correctly except when asked about eating poultry that had been vaccinated against avian influenza. This question also had the highest number of “don’t know” responses. Respondents are most knowledgeable about the preventive measure of culling chickens, perhaps because of the media attention these events attract. The large percentage of correct answers for some questions points to successes of previous information campaigns and media coverage, but the 40% of respondents answering incorrectly or “don’t know” to questions about poultry and egg consumption and human-to-human virus transmission leaves areas for further work.

**Table:** Knowledge of human risks associated with avian influenza, European region, 2006*

For each of the following statements tell me whether, in your opinion, it is true or false:			
Transmission risks	Response, no. (%)		
	True	False	Don’t know
The avian influenza virus can be transmitted between humans	9,864 (33.8)	**16,574 (56.8)**	2,732 (9.4)
Humans can catch avian influenza by touching contaminated birds	**22,722 (77.9)**	4,473 (15.3)	1,975 (6.8)
Food-related risks			
Even when it is contaminated, poultry is not a health risk if it is cooked	**17,906 (61.4)**	8,536 (29.3)	2,728 (9.4)
The avian influenza virus contained in an egg or present on its shell can be eliminated by prolonged cooking	**17,369 (59.5)**	6,593 (22.6)	5,208 (17.9)
It is not dangerous to eat the meat of a chicken vaccinated against avian influenza	**12,833 (44.0)**	9,272 (31.8)	7,065 (24.2)
Other			
The vaccination against seasonal influenza is also effective against avian influenza	4,265 (14.6)	**20,847 (71.5)**	4,058 (13.9)
If a chicken is contaminated by avian influenza on a farm, all the poultry on that farm must be destroyed immediately	**24,492 (84.0)**	2,725 (9.3)	1,953 (6.7)

*Source: Eurobarometer 65.2 (http://ec.europa.eu/public_opinion/archives/eb/eb65/eb65_ee_exec.pdf). Information in **boldface** refers to the correct answer.

These results support previous findings that knowledge about avian influenza, especially about prevention and human-to-human transmission, has scope for improvement (*4*,*5*). Persons in Europe reported that they have little ability to prevent themselves from getting avian influenza (*6*). Previous research in the Lao People’s Democratic Republic examined how consumers’ knowledge of avian influenza risk reduced the likelihood that consumers will substitute poultry for other foods during an avian influenza crisis. This research indicates the importance of informing persons about consumption and transmission-related risks to reduce the likelihood of unnecessary behavioral changes that can cause larger macrolevel market effects (*7*).

The state of knowledge about avian influenza in Europe during the outbreak in the spring of 2006 leaves room for further public health information campaign efforts, especially those that increase consumers’ understanding of consumption-related avian influenza risks. Persons in Europe appear to be aware of culling procedures and the risks of touching infected birds but have a more limited understanding of how avian influenza in their region should influence their consumption patterns.
